# Randomized clinical trial to compare the efficacy of self-expanding bare metal nitinol stent and balloon angioplasty alone for below-the-knee lesions following successful balloon angioplasty: 1-year clinical outcomes

**DOI:** 10.1371/journal.pone.0294132

**Published:** 2023-11-13

**Authors:** Jihun Ahn, HyeYon Yu, Seung-Woon Rha, Byoung Geol Choi, Dong Oh Kang, Cheol Ung Choi, Sangho Park, Jon Seo, Kichang Kim, Minung Kim, Yong Hoon Kim, Yong Seong Seo

**Affiliations:** 1 Department of Internal Medicine, Daejeon Eulji Medical Center, Eulji University School of Medicine, Daejeon, Korea; 2 School of Nursing, College of Medicine, Soonchunhyang University, Asan, Korea; 3 Cardiovascular Center, Korea University Guro Hospital, Seoul, Korea; 4 Department of Internal Medicine, Soonchunhyang University Hospital, Cheonan, Korea; 5 Department of Internal Medicine, Soonchunhyang University Hospital, Bucheon, Korea; 6 Department of Internal Medicine, Shihwa General Hospital, Siheung, Korea; 7 Department of Internal Medicine, Changwon Hanmaeum Hospital, Changwon, Korea; 8 Department of Internal Medicine, Kangwon National University School of Medicine, Chuncheon, Korea; 9 Department of Internal Medicine, Myongji Hospital, Goyang, Korea; Kurume University School of Medicine, JAPAN

## Abstract

This prospective, multicenter, randomized study aimed to compare the 1-year clinical outcomes after primary stenting with self-expanding bare metal nitinol stent (SENS) and plain old balloon angioplasty (POBA) in patients with critical limb ischemia (CLI) and below-the-knee (BTK) lesions. Overall, 119 patients with CLI and BTK lesions were randomized to POBA alone (POBA group, 61 patients) or primary stenting with SENS (stenting group, 58 patients) after achieving acceptable POBA results in target BTK lesions. Clinical outcomes including amputation and revascularization rates were prospectively compared for 1 year. After 1 year, similar incidence rates of individual clinical endpoints, including cardiac death (6.5% vs. 5.1%, p > 0.999), myocardial infarction (1.6% vs. 0.0%, p > 0.999), repeat revascularization (19.6% vs. 18.9%, p = 0.922), target lesion revascularization (13.1% vs. 17.2%, p = 0.530), and amputation (4.9% vs. 0.0%, p = 0.244), were observed. POBA appeared to have acceptable treatment outcomes compared with primary stenting with SENS after 1 year in CLI patients with BTK lesions undergoing percutaneous transluminal angioplasty (PTA).

## Introduction

With the advent of newer techniques and growing evidence, endovascular intervention is a frontline treatment strategy for treating below-the-knee (BTK) lesions with critical limb ischemia (CLI) [1–3]. However, there is no general consensus regarding strategies and treatment outcomes of BTK lesions with CLI. Recently, several interventional treatment strategies, such as plain old balloon angioplasty (POBA) and stenting (primary or bail-out) using bare-metal stents (BMS) or drug-eluting stents (DES), have been evaluated for treating BTK lesions with CLI [4–7]. Furthermore, some studies have reported the effectiveness of self-expanding bare metal nitinol stent (SENS) implantation as a bail-out strategy [8]. Despite these numerous studies, there is still a lack of well-designed multicenter randomized studies comparing the efficacy between primary stenting with SENS and POBA. Thus, we designed this multicenter randomized prospective study to compare the therapeutic effects of primary stenting with SENS and POBA for treating CLI with BTK lesions.

## Methods

This randomized clinical trial was conducted in 18 well-experienced tertiary endovascular intervention centers in South Korea to compare the efficacy of SENS implantation to balloon angioplasty alone for BTK lesions following successful balloon angioplasty (SENS-BTK trial). The study was conducted in accordance with the ethical guidelines of the 2004 Declaration of Helsinki. Institutional Review Board (IRB) of a Korea University Guro Hospital (KUGH) approved all of the consenting procedures. All patients or their legal guardians were given a thorough written and verbal explanation of the study procedure before obtaining written consent for the participation in this study. The authors of this manuscript have certified that the information contained herein is true and correct as reflected in the records of the IRB (#KUGH MD120008). Angiographic images and data management were analyzed by the Cardiovascular Intervention Research Institution, a core laboratory independent of the sponsor (Abbott Vascular, Redwood City, Santa Clara, California, USA).

### Patient selection and study design

This trial compared the clinical outcomes of primary stenting and POBA for the treatment of patients with CLI and BTK lesions. Between December 2012 and September 2016, patients with CLI and BTK lesions were enrolled in this prospective, multicenter, randomized trial. Patients were eligible if they had peripheral arterial disease with ischemic rest pain, wounds, such as ulcers or gangrene, that manifested as BTK lesions. More than half of included patients had wounds with a Rutherford classification (RC) of 4–6. If the wound site was related to a vascular origin according to the angiosome concept, patients were included regardless of the RC of their wound. Patients with acute limb ischemia, those with a life expectancy < 1 year, systemic coagulopathy, or intolerance to antiplatelet agents were excluded. Angiographic eligibility criteria included the presence of de novo stenotic lesions involving 50% of the lumen or occlusion of BTK arteries, and patients meeting these criteria were considered acceptable for endovascular therapy (2.0–4.5 mm in diameter, lesion length < 80 mm owing to the stent length).

### Randomization

**Fig 1:**
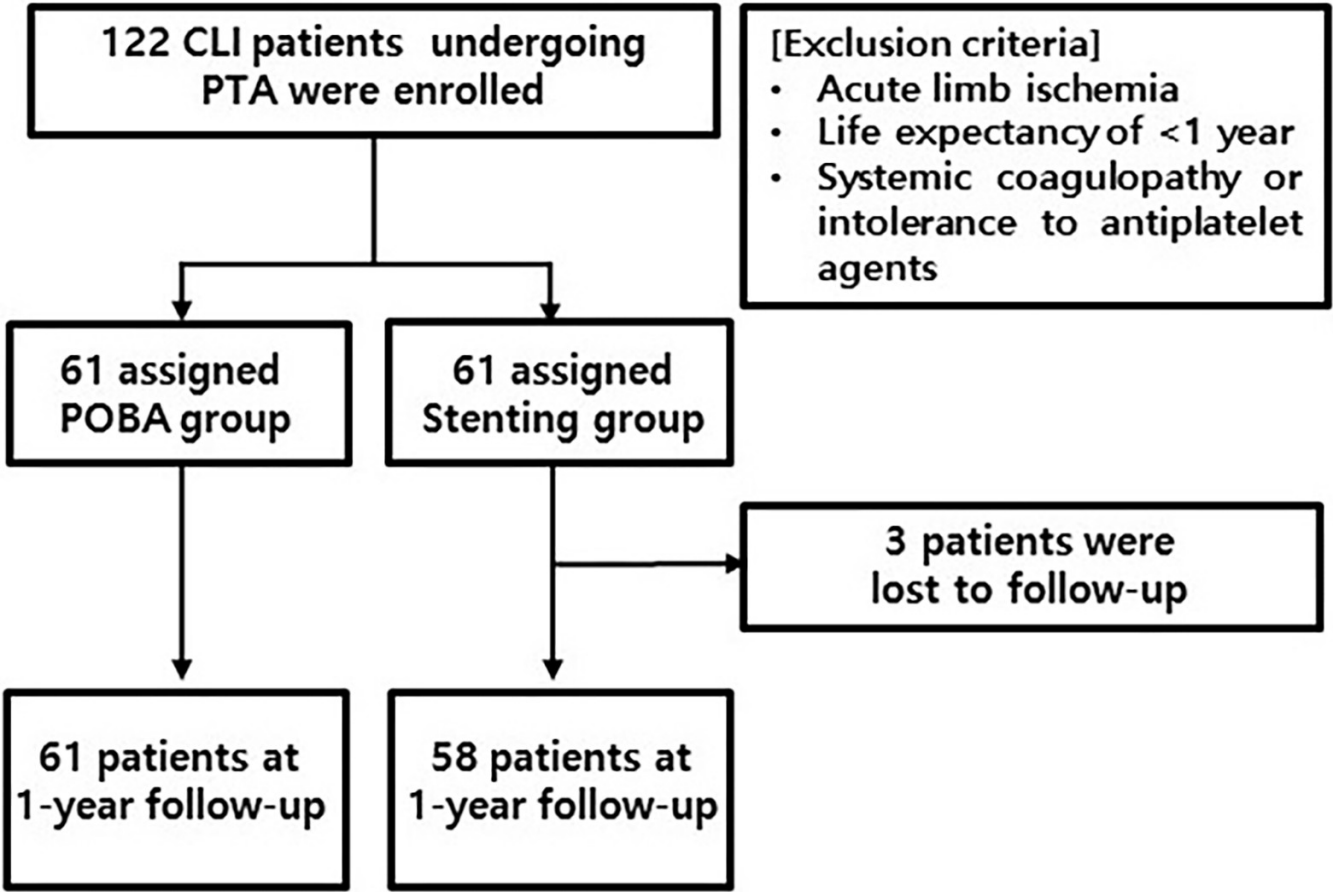
Flow chart.

Eligibility was assessed, and informed consent was obtained by the physicians. Randomization was performed in a 1:1 ratio using a computer-generated randomization sequence so that all patients enrolled in this study were randomized to undergo either SENS implantation (stenting group) or POBA alone (POBA group). For a fair comparison, patients who achieved satisfactory results with balloon angioplasty were randomly assigned to either the POBA group or the stenting group. The POBA group ended the procedure after randomization and the stenting group received SENS implantation. To compare groups in which the lesions had a homogeneous character, only a single target lesion < 80 mm in length in each leg of each patient was selected and treated with an individual stent or POBA alone. Between December 2012 and September 2016, 119 patients were included in this study. In total, 61 patients were randomly assigned to undergo POBA alone, and 58 patients were randomly assigned to undergo SENS implantation (Fig 1). Also, 3 patients in the stenting group were excluded due to follow-up loss.

### Intervention and antiplatelet regimen

All patients were administered loading doses of clopidogrel (300–600 mg) and aspirin (200–300 mg) before the procedure. After sheath insertion at the arterial access site, a bolus dose of unfractionated heparin (70–100 units/kg) was administered. All patients received aspirin (100 mg daily) and clopidogrel (75 mg daily) as a dual antiplatelet (DAPT) maintenance regimen for at least 1 month. Cilostazol (100 mg bid or 200 mg dp daily) was prescribed based on the physician’s discretion as a triple antiplatelet on top of clopidogrel-based DAPT. Some physicians used cilostazol-based DAPT 3–6 months after the index procedure to reduce restenosis.

PTA was performed using the standard technique. After successfully crossing the target lesion with a 0.014-inch guidewire, either intraluminally or subintimally, prolonged balloon dilatation with an adequately sized balloon (1–1.1:1 balloon-to-artery ratio) was performed for 2–3 min. Some patients were recanalized via the retrograde or transcollateral approach following unsuccessful reentry into the distal true lumen using an antegrade approach. To minimize crossover from PTA to stent implantation, prolonged balloon dilatation was performed in patients with unsatisfactory results for balloon dilatation. Once the POBA results were acceptable and the procedure was completed, randomization was performed to leave it alone or with additional stenting. In the stenting group, Xpert (Abbott Vascular, Abbott Park, IL, USA) SENSs were used for routine primary stenting.

### Study endpoints

The main study end-point was the target extremity amputation rate of the patient groups at 1 year. Imaging follow-up was intended to be an additional study endpoint. However, patient characteristics and limitations of insurance coverage in South Korea with CLI made routine follow-up with imaging studies difficult. In addition, limb salvage is considered the most important target of revascularization in patients with CLI. Hence, to assess more clinically relevant information, target extremity amputation was set as the primary endpoint, revascularization including TLR at 1 year, was set as the secondary endpoint.

Death, including cardiac death, myocardial infarction (MI), stroke, and wound recurrence were also assessed during follow-up. Follow-up visits were planned at 1, 6, and 12 months after the index procedure; clinical examinations, laboratory investigations, and imaging studies were performed according to the physician’s advice. One year after the index procedure, follow-up data were collected via face-to-face interviews at the outpatient clinic, review of medical records, and/or telephone conversations with the patients.

### Statistical analyses

Data for all endpoints were evaluated using intention-to-treat analysis. All data are expressed as mean ± standard deviation. The unpaired Student’s t-test and Mann–Whitney rank test were used to compare continuous variables. Categorical variables were compared using the chi-squared and Fisher’s exact tests. The Kaplan-Meier method with a log-rank test was used to compare the cumulative incidence of primary and secondary endpoints. A p value < 0.05 was considered statistically significant. All statistical analyses were performed using the SPSS software (version 20.0, SPSS Inc., Chicago, IL, USA).

## Results

### Baseline clinical and angiographic characteristics of patients, limbs, and lesions

The baseline clinical and laboratory characteristics are presented in Table 1. The results were similar between the two groups. The baseline clinical characteristics of the patients’ limbs are presented in Table 2. There were no differences between the two groups in terms of RC or wound site. The incidence rates of minor and major tissues loss were 59% and 0% in the POBA group and 68.9% and 0% in the stenting group, respectively. The ankle-brachial index (ABI) was numerically lower in the POBA group than in the stenting group; however, the difference was statistically insignificant (0.79 ± 0.38 vs. 0.93 ± 0.29, p = 0.105). The characteristics of the limbs and lesions are presented in Table 3. There were no significant differences between the two groups in terms of the location of the lesion and combined inflow arterial disease. The size and length of the balloon were numerically larger in the POBA group than in the stenting group, although the difference was not statistically significant.

**Table 1:** Baseline clinical and laboratory characteristics.

Variables, N (%)	POBA group (n = 61)	Stenting group (n = 58)	P value
**Sex, male**	54 (88.5)	46 (79.3)	0.170
**Age, year**	66.5 ± 9.6	68.8 ± 9.0	0.259
**Body mass index, Kg/m** ^**2**^	22.9 ± 3.9	23.1 ± 3.6	0.690
**Hypertension**	42 (68.8)	35 (60.3)	0.332
**Diabetes mellitus**	49 (80.3)	49 (84.4)	0.552
** Medication**	39 (63.9)	37 (63.7)	0.987
** Insulin**	12 (19.6)	18 (31.0)	0.154
**Chronic renal insufficiency**	19 (31.1)	12 (20.6)	0.194
** End-stage renal disease**	12 (19.6)	9 (15.5)	0.552
**Heart failure**	4 (6.5)	2 (3.4)	0.680
**Dyslipidemia**	3 (4.9)	4 (6.8)	0.713
** Previous use of statin**	12 (19.6)	6 (10.3)	0.156
**Tobacco**	22 (36.0)	20 (34.4)	0.857
** Currently**	14 (22.9)	15 (25.8)	0.712
** Quit**	8 (13.1)	5 (8.6)	0.432
**Coronary artery disease**	17 (27.8)	14 (24.1)	0.643
**Stroke**	10 (16.3)	7 (12.0)	0.500
** Hemorrhagic**	1 (1.6)	1 (1.7)	>0.999
** Ischemic**	8 (13.1)	3 (5.1)	0.135
** TIA**	1 (1.6)	3 (5.1)	0.356
**Lab findings**			
**Hemoglobin (g/dL)**	11.3 ± 2	11.5 ± 1.7	0.584
**Hematocrit (%)**	33.7 ± 5.9	34.2 ± 5.1	0.618
**Glucose (mg/dL)**	175 ± 108	186 ± 122	0.598
**HbA1c (%)**	7.4 ± 2.0	7.8 ± 2.1	0.377
**Creatinine (mg/dL)**	2.74 ± 3.05	1.98 ± 1.85	0.793
**Total Cholesterol (mg/dL)**	136 ± 36	143 ± 39	0.582
**Triglyceride (mg/dL)**	128 ± 57	133 ± 80	0.673
**HDL-C (mg/dL)**	35 ± 10	35 ± 12	0.996
**LDL-C (mg/dL)**	86 ± 29	79 ± 31	0.268
**hs-CRP (mg/L)**	8.29 ± 15.13	6.92 ± 8.07	0.958
**Medications**			
**Aspirin**	57 (93.4)	57 (98.2)	0.365
**Clopidogrel**	57 (93.4)	54 (93.1)	>0.999
**Cilostazol**	10 (16.3)	10 (17.2)	0.902
**Other anticoagulants**	12 (19.6)	13 (22.4)	0.714
**Warfarin**	2 (3.2)	2 (3.4)	>0.999
**Prostaglandin**	3 (4.9)	4 (6.8)	0.713
**RAS inhibitors**	24 (39.3)	19 (32.7)	0.455
**Calcium channel blocker**	13 (21.3)	16 (27.5)	0.425
**Beta blocker**	18 (29.5)	10 (17.2)	0.115
**Diuretics**	4 (6.5)	10 (17.2)	0.071
**Statin**	43 (70.4)	43 (74.1)	0.657

POBA, plain old balloon angioplasty; TIA, transient ischemic attack; HbA1c, hemoglobin A1c; HDL, high density lipoprotein; LDL, low density lipoprotein; hs-CRP, high sensitivity c-reactive protein; RAS, renin-angiotensin system.

**Table 2:** Baseline clinical characteristics of the patients’ limbs.

Variables, N (%)	POBA group (n = 61)	Stenting group (n = 58)	P value
Target limb			
** **Right	32 (52.4)	26 (44.8)	0.405
** **Left	28 (45.9)	31 (53.4)	0.411
Ankle-brachial index	0.79 ± 0.38	0.93 ± 0.29	0.105
TcPO2 (Wounded only)	30.2 ± 20.9	15 ± 4.2	0.245
Rutherford class			0.284
** **4, Ischemic rest pain	25 (41.0)	17 (29.3)	
** **5, Minor tissue loss	36 (59.0)	40 (69.0)	
** **6, Major tissue loss	0 (0.0)	1 (1.7)	
Wound site			
** **Toe, dorsal side	33 (54.0)	29 (50.0)	0.655
** **Toe, plantar side	27 (44.2)	24 (41.3)	0.751
** **Sole of the foot	9 (14.7)	6 (10.3)	0.469
** **Dorsum of the foot	8 (13.1)	5 (8.6)	0.432
** **Heel	3 (4.9)	6 (10.3)	0.315
** **Ankle	0 (0.0)	2 (3.4)	0.235
** **Leg	1 (1.6)	0 (0.0)	>0.999

POBA, plain old balloon angioplasty; TcPO2, transcutaneous oxygen pressure.

**Table 3:** Baseline angiographic and clinical characteristics of the patients’ lesions and limbs during procedures.

Variables, N (%)	POBA group (n = 61)	Stenting group (n = 58)	P value
**Stenosis (>50%)**			
** Aorta**	1 (1.6)	0 (0.0)	>0.999
** Common iliac arteries**	7 (11.4)	3 (5.1)	0.324
** External iliac arteries**	1 (1.6)	3 (5.1)	0.356
** Common femoral artery**	1 (1.6)	1 (1.7)	>0.999
** Superficial femoral artery**	22 (36.0)	20 (34.4)	>0.999
** Popliteal artery**	4 (6.5)	6 (10.3)	0.522
** Anterior tibial artery**	44 (72.1)	42 (72.4)	>0.999
** Posterior tibial artery**	40 (65.5)	34 (58.6)	0.456
** Peroneal artery**	29 (47.5)	24 (41.3)	0.581
**PTA**			
** Aorta**	1 (1.6)	0 (0.0)	>0.999
** Common iliac arteries**	5 (8.1)	2 (3.4)	0.440
** External iliac arteries**	0 (0.0)	1 (1.7)	0.487
** Superficial femoral artery**	19 (31.1)	20 (34.4)	0.845
** Popliteal artery**	3 (4.9)	5 (8.6)	0.484
** Anterior tibial artery**	44 (72.1)	42 (72.4)	>0.999
** Posterior tibial artery**	36 (59.0)	28 (48.2)	0.273
** Peroneal artery**	21 (34.4)	19 (32.7)	>0.999
**PTA in BTK lesion**			
**Balloon inflation**			
** Diameter, mm**	3.15 ± 3.56	2.76 ± 0.45	0.442
** Length, mm**	83 ± 71.8	69.4 ± 61.4	0.569
** Pressure, atm**	11.5 ± 4.6	10.9 ± 3.6	0.701
** Time, seconds**	115.6 ± 53.1	91.7 ± 49.4	0.019
**Number of stents**		1.2 ± 0.13	<0.001
**Stent diameter, mm**		3.68 ± 0.72	<0.001
**Stent length, mm**		42.2 ± 15.9	<0.001

POBA, plain old balloon angioplasty; PTA, percutaneous transluminal angioplasty; BTK, below-the-knee.

### One-year clinical outcomes

**Fig 2:**
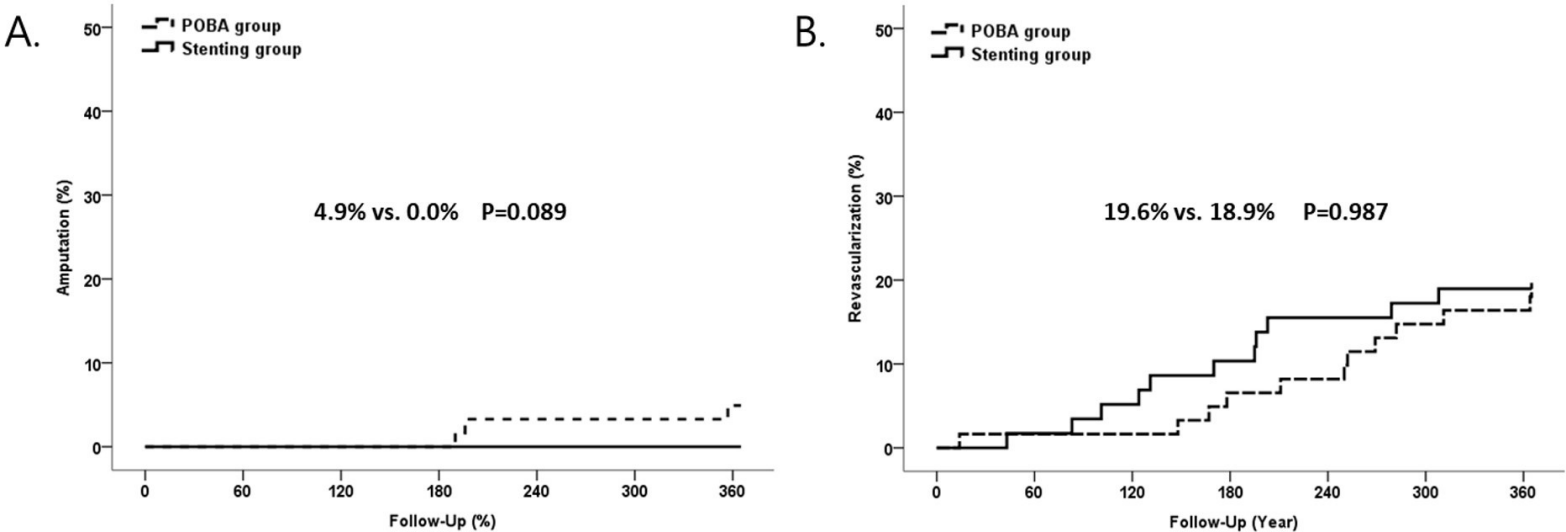
The cumulative incidence of target extremity amputation and revascularization up to 1-year by Kaplan-Meier analysis.

A clinical investigation was performed on all patients at the 1-year follow-up visit. The 1-year clinical outcomes are presented in Table 4. The primary endpoint, target extremity amputation rate (4.9% vs. 0%, p = 0.244), and the secondary endpoint, revascularization rate (19.6% vs. 18.9%, p = 0.922), showed no significant difference between the groups (Fig 2). Other clinical events, including death, MI, stroke, bleeding, and wound recurrence, were also assessed, and the results were similar between the groups.

**Table 4:** One-year clinical outcomes.

Variables, N (%)	POBA group (n = 61)	Stenting group (n = 58)	P value
**Death**	5 (8.1)	7 (12.0)	0.483
** Cardiac death**	4 (6.5)	3 (5.1)	>0.999
**MI**	1 (1.6)	0 (0.0)	>0.999
**Stroke**	1 (1.6)	2 (3.4)	0.612
**Peripheral arterial occlusive disease**	17 (27.8)	17 (29.3)	0.862
**Wound**	7 (11.4)	8 (13.7)	0.703
**Revascularization**	12 (19.6)	11 (18.9)	0.922
** TLR**	8 (13.1)	10 (17.2)	0.530
** Non-TLR**	2 (3.2)	1 (1.7)	>0.999
** TVR**	9 (14.7)	10 (17.2)	0.711
** Non-TVR**	3 (4.9)	1 (1.7)	0.619
** Target extremity revascularization**	9 (14.7)	10 (17.2)	0.711
**Bleeding**	2 (3.2)	1 (1.7)	>0.999
** Major**	0 (0.0)	1 (1.7)	0.487
** minor**	2 (3.2)	1 (1.7)	>0.999
**Amputation**	3 (4.9)	0 (0.0)	0.244
** Major (above the ankle)**	1 (1.6)	0 (0.0)	>0.999
** Minor (below the ankle)**	3 (4.9)	0 (0.0)	0.244

POBA, plain old balloon angioplasty; MI, myocardial infarction; TLR, target lesion revascularization; TVR, target vessel revascularization.

## Discussion

The main findings of this study were as follows. In patients with CLI with a BTK lesion undergoing an intervention, (1) clinical outcomes, including amputation and cardiovascular event rates, were similar between the primary stenting and POBA groups; (2) amputation and revascularization rates were relatively low in both groups; and (3) reasonable results could be derived only from short-term clinical follow-up, and imaging follow-up was not compulsory.

With the rapid improvement in device technology along with increasing operator experience, interventional treatment is a well-accepted procedure in patients with a BTK lesion and CLI [9–11]. Multiple studies have investigated the clinical outcomes of SENS implantation using Xpert stents and compared SENS implantation with POBA. However, most of these studies were nonrandomized, restricted to short-length disease, and single-center or single-arm trials [8, 12]. Therefore, an accurate and well-designed comparison between primary stenting with SENS implantation and POBA in treating of CLI has not been performed. This prospective randomized multicenter study demonstrates acceptable clinical outcomes for POBA compared with primary stenting with SENS implantation in the treatment of patients with BTK lesions and CLI in a well-organized and relatively homogenous study population.

The most critical limitations of previous studies on patients with CLI were the format of the study and the heterogeneous strategies, including primary and bail-out stenting in patients undergoing stenting with BTK lesions [13–15]. In this study, to overcome this methodological weakness, we compared primary stenting using SENS with POBA alone. All patients included in this study had CLI during the recruitment period, and interventional treatment through primary stenting using SENS was thoroughly applied to the group classified as the stenting group.

CLI with a BTK lesion is usually an extensive and multilevel arterial disease that results in adverse clinical outcomes [16]. SENS has a mechanism that has a theoretical advantages of flexibility, super-elasticity, biocompatibility, radial force, and shape memory effect of the vascular wall in the treatment of complex BTK lesions [17–19]. The XCEEL study reported a 12-month amputation-free survival rate of 78.3% and target lesion revascularization (TLR) rate of 29.9%. However, on the contrary, the EXPAND study reported no differences in terms of freedom from TLR, amputation, and mortality rates between primary stenting and bail-out stenting techniques [20–23]. Although previous studies have reported mixed results regarding primary stenting, the present study indicated that the frequency of recanalization of blood vessels and 1-year cardiovascular endpoints, such as cardiac death (6.5% vs. 5.1%) and MI (1.6% vs. 0%), were low and comparable in both the primary stenting and PTA groups. The most important finding of this study was that the major amputation rate was 0%, which indicates a high limb salvage rate. Thus, we conclude that both POBA and primary stenting can be adopted as interventional treatment options for patients with BTK lesions and CLI.

In this study, the primary stenting (0.0%) and POBA (1.6%) groups showed a low major amputation rate. Even with the use of advanced techniques, the development of an interventional method alone is insufficient to explain the significantly low limb recovery rates demonstrated in both groups. The development of wounds and CLI are related to diabetes [24]. Additionally, the aggravation of wounds is caused by issues with vessel patency and by the combined effects of hyperglycemia and neurological, vascular, and immunological factors. A multidisciplinary treatment approach toward wound care and diabetic control is important [25]. A collaboration with the other department such as surgical and endocrinological department can improve a patient’s clinical prognosis by performing procedures, such as debridement, grafting, local flap, stem cell therapy, minor amputation, careful dressing and active glucose control before and after index procedures.

This study also elucidated the importance of setting an appropriate endpoint for the treatment of BTK lesions with CLI. In the coronary era, clinical practices have shifted toward minimizing the use of routine imaging tests and focusing on clinical symptoms, and this has resulted in good patient outcomes [26]. In patients with CLI, outcomes are affected by multiple heterogeneous factors, such as patient-, external-, and functional-related variables, including diabetes, level of medical care units, and patient’s general condition [27]. Therefore, instead of evaluating a patient’s prognosis for a fragmentary purpose and adopting a routine angiographic follow-up, it is reasonable to use a patient-centric outcome as the endpoint of the study. This approach has been adopted by other studies as well [13, 20]. Clearly, imaging tests and ABI play an important role in diagnosing and treating peripheral vascular disease [28]. However, this study focused on the patient’s clinical prognosis with a “patient-centric” approach with selective imaging study follow-up. It showed a low amputation rate in both the groups independent of the repeat revascularization rate (19.6% in the POBA group vs. 18.9% in the stenting group).

This study had some limitations. First, it addressed only BMS and POBA alone. However, there are several newer devices, such as drug-coated balloons, DES, and polymer-free stents, and a study comparing these devices should be conducted. Second, the relatively small sample size and short duration of the study period did not provide strong evidence of the actual clinical efficacy and safety of the procedures. Third, although this was a multicenter study and the general goal of CLI treatment was early-phase ulcer healing, large-scale and long-term clinical investigations are required. Fourth, this study was designed to focus on a “patient-centric” concept; hence, follow-up imaging modalities, such as computed tomography angiography and conventional angiography, were not routinely performed. These investigations were only performed in the event of wound recurrence or at the physician’s discretion, regardless of ischemic symptoms. Even if restenosis occurred, follow-up imaging tests may not have been performed if the wound did not recur or patients did not complain of definite claudication during the follow-up period after the index procedure. Thus, the actual restenosis rate may not have been reflected. Finally, due to the characteristics of the lesions in the patients with CLI, there were patients with iliac artery or femoral artery lesions [29]. The study was not conducted solely for patients with infra-popliteal artery disease. Despite these limitations, this study prospectively investigated and randomly enrolled patients with CLI. However, long-term studies with a larger number of patients and routine follow-up imaging tests are required to reach definitive conclusions.

## Conclusions

In this prospective randomized multicenter study, during the 1-year follow-up, primary stenting with SENS and POBA alone demonstrated a significantly low rate of clinical adverse events in treating patients with CLI and BTK lesions. These results indicate that POBA may be an acceptable option in treating CLI with BTK lesions avoiding stent implantation. Also, based on the non-inferior results of the stenting group, SENS implantation could remain the preferred treatment option in special circumstances such as persistent recoil and flow-limiting dissection despite prolonged balloon dilatation in the treatment of CLI with BTK lesions. Long-term follow-up studies with larger study populations and routine imaging follow-up tests are necessary to elucidate the final conclusions.

## Supporting information

S1 Checklist Reporting checklist for randomised trial. (DOCX)

S1 Protocol SENS-BTK prospective clinical study protocol. (DOC)
