# Supplementary material for: Randomized clinical trial to compare the efficacy of self-expanding bare metal nitinol stent and balloon angioplasty alone for below-the-knee lesions following successful balloon angioplasty: 1-year clinical outcomes
Source: PLoS One. 2023 Nov 13;18(11):e0294132. doi: 10.1371/journal.pone.0294132 (PMC10642822; doi:10.1371/journal.pone.0294132)
Supplement: S1 Protocol — (DOC) [file pone.0294132.s002.doc]

**SENS-BTK Prospective Clinical Study Protocol**

1. **Study Title**

Efficacy of **S**elf-**E**xpanding **N**itinol **S**tent versus Balloon Angioplasty Alone for the **B**elow **T**he **K**nee Arteries following Successful Balloon Angioplasty Trial (**SENS-BTK** trial): Korean Prospective Randomized Multicenter Clinical Trial

1. **Backgrounds**

While endovacular interventions (EVT) and clinical research for peripheral vascular diseases (PVD) of the iliac and femoropopliteal arteries are common, there are relatively limited number of EVT and clinical research for the below-the-knee (BTK) arteries. Nowadays, the interests for the BTK intervention is rapidly increasing due to increased prevalence of diabetes mellitus (DM) world widely and active screening program in high volume centers. Recently, favorable outcomes following EVT have been reported in critical limb ischemia patients, especially those with diabetes as comorbidity or who are not eligible for bypass surgery.

Percutaneous transluminal balloon angioplasty (PTA) has been increasingly performed over the past decades since small-sized profile catheters, hydrophilic guide wires, balloons, stents and other innovative devices were introduced. Also in the infrapopliteal arteries, stenting is applied in case that it has been failed to restore anterograde flow even after the balloon angioplasty with a conventional endovascular intervention technique. In the past, stents for the below-the-knee vessels only was not introduced in South Korea; however, self-expanding nitinol stent for such the small infrapopliteal vessels (Xpert Stent, AbbottVascular, San Francisco, Calif., USA) has become commercially available in recent days.

According to a report of the outcomes of conventional balloon angioplasty for the infrapopliteal arteries in patients with critical limb ischemia, the procedural success rate was estimated at 93%; restenosis, revascularization and lower limb amputation rates at 12 months 39%; vascular patency rate at 12 months 53%; and, as secondary endpoints, restenosis and revascularization rates at 12 months 63% and 61%, respectively. It was also reported that limb salvage rate was 84% at 12 months (J Vasc Interv Radiol 2007; 18:703–708). Although the conventional balloon angioplasty exhibits approximately 90% of good limb salvage rate as described above, it shows actually unsatisfactory primary vascular patency rates and more unfavorable outcomes in long and complex lesions.

In addition, it is reported that the failure rates of conventional balloon angioplasty for the infrapopliteal arteries range from 10% to 40% depending on lesion type and surgeon’s experience. Besides, in case of severe calcification, residual stenosis ≥50%, dissection leading to blood flow disturbances, or occlusive thrombus formation not responsive to thrombolysis, thrombus aspiration or balloon angioplasty, stenting is usually attempted following balloon angioplasty. Because of concern about early thrombus formation resulting from stent placement in the small-diameter infrapopliteal arteries and possible late lumen loss caused by intimal hyperplasia, routine stenting in the infrapopliteal arteries are not being widely spread.

The self-expanding nitinol stent for the below-the-knee arteries were developed for the purpose of maintaining stable blood flow, preventing lumen loss and ultimately improving vascular patency rate. When the self-expanding stent was implanted in 16 patients with critical limb ischemia who were not respond to conventional balloon angioplasty, the vascular patency rate at 6 months was obtained from 14 patients of them, and good patency was observed in 12 lesions.

In a study of below-the-knee artery balloon angioplasty followed by routine (self-expanding) stenting in 47 patients with critical limb ischemia as it was expected that it would contribute significantly to good clinical outcomes, it was found that the vascular patency and limb salvage rates were 76.3% and 95.9% at 12 months, and 54% and 90.8% at 24 months, respectively

As mentioned above, with the recent advances of medical technologies and the introduction of self-expending stents only for the infrapopliteal arteries, vascular interventions in critical limb ischemia are radically developing. However, the gold standard therapy has not been established yet like that for coronary artery interventions. Moreover, there is currently a lack of comparative studies of balloon angioplasty alone *versus* balloon angioplasty with routine stenting – the former is that it is left as it is in the case of good responses to balloon angioplasty and the latter is that stenting is routinely conducted after balloon PTA, similarly to coronary artery intervention.

In the context above, the present investigators attempted to identify the effects of conventional balloon angioplasty with self-expanding nitinol stent in the occlusive lesions of the infrapopliteal arteries in patients with critical limb ischemia, in the manner of collecting case data and analyzing comparatively patient groups treated with balloon angioplasty alone versus balloon angioplasty with routine stenting in a prospective multicenter randomized clinical trial.

1. **Study Objectives**

The objectives of this study are to compare directly conventional balloon angioplasty alone *versus.* balloon angioplasty with routine stenting – that is, to determine whether angioplasty with self-expanding stent is superior to conventional balloon angioplasty - in the infrapopliteal arterial occlusive lesions of critical limb ischemia patients by collecting and analyzing the cases of each patient group in a prospective multicenter randomized clinical trial, and to clarify main factors affecting mid- and long-term clinical effects of angioplasty with self-expanding stent in the infrapopliteal arteries.

Hypothesis: Balloon PTA followed by routine stenting with self-expanding nitinol stent in critical limb ischemia patients with infrapopliteal arterial occlusive lesions is superior to conventional PTA followed by provisional stenting, in the aspect of vascular restenosis rate.

1. **Study Sites and Period:**

Korean, national, multicenter prospective randomized clinical trial

Study Period: From Sept 2011 to July 2014

**Participating centers:**

|  | Participating centers (sites) | Principal investigators | Co-investigators |
| --- | --- | --- | --- |
| **1** | Korea University Guro Hospital | Seung-Woon Rha | Cheol-Ung Choi |
| **2** | Kwandong University MyoungJi Hospital | Yun-Hyeong Cho |  |
| **3** | KonKuk University Chungju Hospital | Woonggil Choi |  |
| **4** | Soonchunhyang University Cheonan Hospital | Sang-Ho Park | Seung Jin Lee |
| **5** | Yonsei University Shinchon Severance Hospital | Young-guk Ko | Dong Hoon Choi |
| **6** | Yonsei University Gangnam Severance Hospital | Pil Ki Min |  |
| **7** | KyungHee University Medical Center | Kim, Won |  |
| **8** | Gachon University Gil Hospital | Tae-Hoon Ahn | Woong Cheol Kang |
| **9** | Sam Medical Center, AnYang | Seong Gyu Yoon | Yun Kyung Kim |
| **10** | SamSung Medical Center | Seung Hyuk Choi |  |
| **11** | Eulji University Hospital | Won Ho Kim |  |
| **12** | Sejong General Hospital | Cheol Woong Yoo | Won Heum Shim |
| **13** | Incheon Sarang Hospital | Ki Chang Kim |  |
| **14** | Pusan National University Hospital | Han Cheol Lee |  |

1. **Inclusion/Exclusion Criteria**

*a. Inclusion criteria*: All of the criteria listed below shall be met.

1. Clinical criteria
   1. Age 20 years of older
   2. Symptomatic critical limb ischemia (Rutherford 4 - 6)
   3. Patients with signed informed consent
2. Anatomical criteria
   1. Target lesion length < 8 cm by angiographic estimation
   2. Stenosis of >50% or occlusive atherosclerotic lesion of the ipsilateral infrapopliteal artery
   3. Reference vessel diameter should be 2.0–4.5 mm.

*b. Exclusion criteria:*

1. Clinical criteria
   1. Patient has a known allergy to heparin, aspirin, or other anticoagulant/antiplatelet therapies or a bleeding diatheses or is unable, or unwilling, to tolerate such therapies
   2. Patient takes warfarin
   3. Patient has a history of previous life-threatening contrast media reaction
   4. Patient is currently enrolled in another investigational device or drug trial
   5. Patient is currently breast-feeding, is pregnant, or intends to become pregnant
   6. Patient is mentally ill or retarded
   7. Acute critical limb ischemia
   8. Major bleeding history within prior 2 months
   9. Age > 80 years
   10. Severe hepatic dysfunction (> 3 times normal reference values)
   11. Serum creatinine level ≥ 3.0 mg/dl or dependence on dialysis
   12. Significant leucopenia, neutropenia, thrombocytopenia, anemia, or known bleeding diathesis
   13. Life expectancy <1 year due to comorbidity
2. Angiographic criteria
   1. Reference segment diameter is not suitable for available stent design
   2. Previously implanted stent(s) or PTA at the same lesion site
   3. Inflow-limiting arterial lesions left untreated
3. **Sample Size Estimation**

On the assumption that the restenosis rate at 12 months after the conventional balloon angioplasty for the infrapopliteal arterial occlusive lesions in patients with critical limb ischemia is estimated approximately at 55%, whether the restenosis rate at 12 months after balloon angioplasty with routine stenting with self-expanding stent is higher than (superior to) that of the conventional balloon angioplasty alone is tested with 80% of power (20% of difference). When computed in ‘Power and Precision V4’ software program to do so, the sample size required is 89 for each group, and in consideration for drop-outs including 30% of follow up loss, the sample size (number of subjects) computed was 116 per group and a total of 232. For the statistical computation method, see the Appendix.

1. **Study Design and Methodology**

- Study design: A prospective multicenter randomized controlled trial

- Study methodology: Critical limb ischemia patients with infrapopliteal arterial occlusive or stenotic lesions at the participating centers between November 2011 and January 2015 are randomly assigned to a group of patients who will undergo provisional stenting following conventional balloon angioplasty and the other group of patients who will undergo routine stenting following conventional balloon angioplasty. Then, for each group, preoperative clinical and lesion characteristics will be investigated at baseline, the intervention procedures, immediate postoperative results, the length of hospital stay, clinical outcomes/course after discharge, and revascularization rates will be evaluated, and the restenosis rate will be angiographically examined at 12 months, followed by analyzing comparatively any factors affecting the rate.


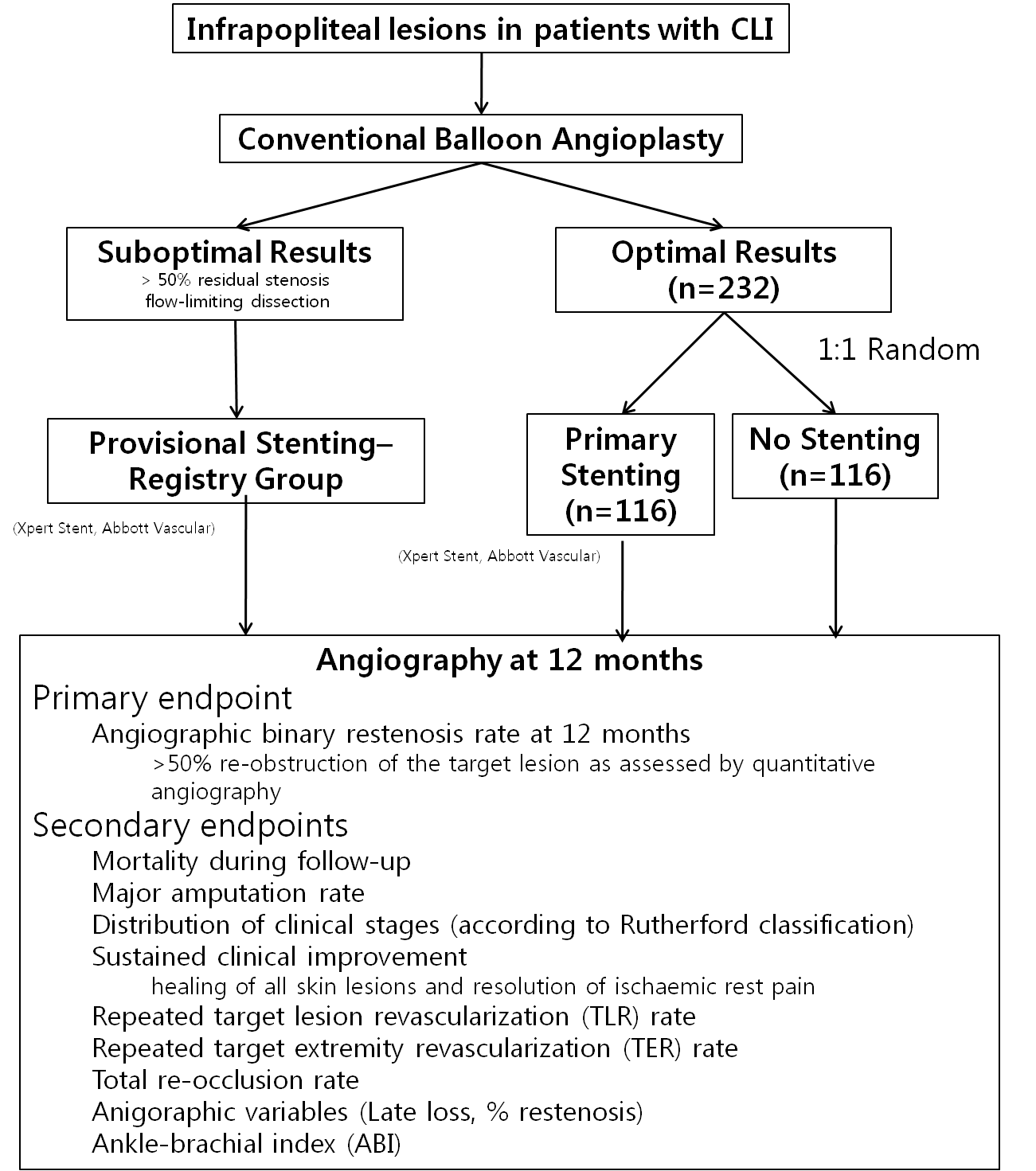


1. **Monitoring/Laboratory and Clinical Parameters**

- Symptom diagnosis: according to Rutherford & Fontaine classification

- Preoperative clinical characteristics at baseline

- Lesion characteristics based upon the findings of imaging

- Intervention procedure related parameters

- Postoperative outcomes/course

1. Imaging parameter (CT, color Doppler): >50% restenosis at 12 months
2. Hemodynamic parameter: Ankle-brachial index, Calf-brachial index, Thigh-brachial index
3. Clinical outcomes/course:
   1. Symptom recurrence or aggravation
   2. Target vessel revascularization
   3. Major cardiovascular adverse events : death, MI, stroke
   4. Lower limb amputation status
4. **Efficacy Evaluation Criteria/Method and Analysis Techniques**

1. Primary endpoint: Binary restenosis at 12 months

Binary restenosis : >50% re-obstruction of the target lesion as assessed by quantitative

angiography

2. Secondary endpoints

1. Target vessel revascularization rate
2. Limb salvage rate: free from amputation
3. Major cardiovascular adverse events : death, MI, stroke
4. Angiographic variables (Binary restenosis, FU MLD, LL, % restenosis)

3. Statistical analysis

Categorical variables will be expressed in percentage, and inter-subgroup comparisons will be analyzed with either Chi-square or Fisher’s exact test. Continuous variables will be expressed in mean ± standard deviation, and inter-subgroup comparisons will be analyzed with Student’s *t* test. In addition, clinical course and outcomes including patient’s symptoms, restenosis assessed based on the findings on ABI and invasive angiograms, target vessel revascularization, major cardiovascular adverse events (death, MI and stroke) and lower limb amputation status will be analyzed with Kaplan-Meier survival estimates, and inter-subgroup comparisons will be analyzed with log-rank test. *p* value <0.05 will be considered statistically significant.

1. **Data Management**

In order to ensure confidentiality of research data, all the personal information of patient data collected will be encoded, recorded and kept so that they cannot be disclosed easily. Additionally, all electronic (computed) data will be saved in a computer to which access will be restricted, and all documentary data will be stored in a locking system.

1. **Study Implementation Plan**

|  | 2011  11 |  |  | 2012  9 |  |  | 2013  8 |  |  |  | 2014  11 | 2015  1 |
| --- | --- | --- | --- | --- | --- | --- | --- | --- | --- | --- | --- | --- |
| Patient enrollment |  |  |  |  |  |  |  |  |  |  |  |  |
| Data collection and analysis |  |  |  |  |  |  |  |  |  |  |  |  |
| Paper Writing |  |  |  |  |  |  |  |  |  |  |  |  |

**References**

1. Giles KA, Pomposelli FB, Spence TL, Hamdan AD, Blattman SB, Panossian H, Schermerhorn ML. Infrapopliteal angioplasty for critical limb ischemia: Relation of TransAtlantic InterSociety Consensus class to outcome in 176 limbs. J Vasc Surg 2008;48:128-36.

2. Kickuth R, Keo HH, Triller J, Ludwig K, Do DD. Initial Clinical Experience with the 4-F Self-expanding XPERT Stent System for Infrapopliteal Treatment of Patients with Severe Claudication and Critical Limb Ischemia. J Vasc Interv Radiol 2007; 18:703–708

3. Bosiers M, Deloose K, Verbist J, Peeters P. Nitinol stenting for treatment of ''below-the-knee'' critical limb ischemia: 1-year angiographic outcome after Xpert stent implantation. J Cardiovasc Surg (Torino). 2007 Aug;48(4):455-61.

4. Tepe G, Zeller T, Heller S, Wiskirchen J, Fischmann A, Coerper S, Balletshofer B, Beckert S, Claussen CD. Self-expanding nitinol stents for treatment of infragenicular arteries following unsuccessful balloon angioplasty. Eur Radiol. 2007 Aug;17(8):2088-95. Epub 2006 Dec 21.

5. Bosiers M, Lioupis C, Deloose K, Verbist J, Peeters P. Two-year outcome after Xpert stent implantation for treating below the knee lesions in critical limb ischemia. Vascular. 2009 Jan-Feb;17(1):1-8.

6. Peregrin JH, Smírová S, Koznar B, Novotný J, Kovác J, Lastovicková J, Skibová J. Self-expandable stent placement in infrapopliteal arteries after unsuccessful angioplasty failure: one-year follow-up. Cardiovasc Intervent Radiol. 2008 Sep-Oct;31(5):860-4.

**Appendix**

**Sample size**

**Executive summary**

With a sample of 89 subjects per group the study will have power of 80%. This means that there is an 80% likelihood that the study will yield a statistically significant effect, and allow us to conclude that the percentage of subjects in 'Response A' differs for Group A versus Group B.

**Details**

The study will compare two groups (Group A versus Group B) on a collection of categories called Categories. The collection is composed of the following 2 categories: Response A and Response B.

Our focus is on the category called 'Response A'. The null hypothesis is that the proportion of subjects in this category is identical in Group A and Group B. Our intent is to disprove the null, and conclude that this proportion is different in the two groups.

The computation of sample size is based on the following assumptions and decisions.

**Group A**

The expected pattern of responses for Group A is as follows (see plot). 'Response A' (55%), 'Response B' (45%). In particular, the percentage in 'Response A' is 55%.


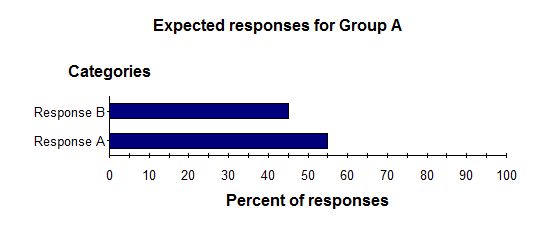


**Group B**

The expected pattern of responses for Group B is as follows (see plot). Response A(75%), Response B(25%). In particular, the percentage in Response A is 75%.


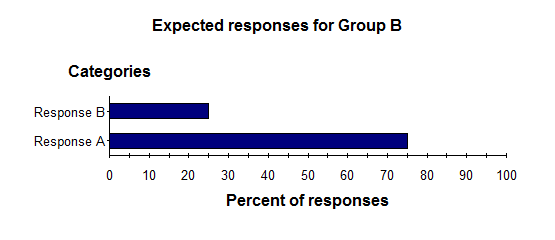


**Missing**

In computing the sample size we assume that there will be no missing data.

**Sample Size**

The study will enroll 89 people per group, for a total of 178 people. With this sample size, there is an 80% likelihood that the study will yield a statistically significant result, and allow us to conclude that the percentage of subjects in 'Response A' is different for Group A than for Group B.

**Understanding the assumptions**

The decision to use a sample size of 89 per group is based on the assumptions outlined above. If these assumptions are correct, then this sample size will result in power of 80%. However, if these assumptions are incorrect, then the sample size needed to yield power of 80% will be higher or lower than 89 per group. Therefore, it is instructive to consider what sample size would be required if we adopted a different set of assumptions.

Computation of the required sample size is based on five factors, as follows.

**Difference between groups**

One factor that determines the required sample size is the mean difference between groups. A small difference is relatively hard to detect, and therefore requires a larger sample size. Conversely, a large difference is relatively easy to detect, and therefore requires a smaller sample size.

The sample size of 89 is based on the assumption that groups differ by 20 percentage points.

**Absolute value of the proportions**

Another factor that determines the required sample size is the absolute value of the proportions. The sample size required to detect a 20 percentage points difference will be larger if the proportions fall near 50%, and will be smaller if the proportions fall near 0% or near 100%.The sample size of 89 is based specifically on the comparison of 55% vs. 75%.

**Missing data**

Another factor that determines the required sample size is the percent of missing data. We compute the number of subjects actually needed for the analysis, and then adjust that number to ensure that we will have that number of responses after the missing subjects are excluded.

In computing the sample size to be 89 we assume that there will be no missing data. If the actual rate of missing data is 2%, we would need a sample size of 90 per group.

Note that the adjustment for missing data assumes that the data are missing completely at random. No attempt is made to adjust for the possibility that people who fail to respond differ in some ways from people who do provide a response.

**Alpha**

Another factor that has an impact on the required sample size is alpha, the criterion used for statistical significance. We used an alpha of 0.05, which is often the default value, in computing the required sample size of 89 per group.

It is sometimes appropriate to select a more conservative criterion. For example, with alpha set at 0.01 the required sample size would be 132 per group. Conversely, it is sometimes appropriate to select a less conservative criterion. For example, with alpha set at 0.10 the required sample size would be 70 per group.

**Tails**

The final factor we need to consider is whether the significance test is one-tailed or two-tailed. We assumed that the study will use a two-tailed test, which is usually appropriate, and computed the required sample size as 89 per group.

If it were appropriate to use a one-tailed test (with alpha at 0.05) the required sample size would be 70 per group.
